# A Comparative Analysis of Serum Irisin Levels and Body Composition Indices in Women With and Without Polycystic Ovary Syndrome (PCOS)

**DOI:** 10.7759/cureus.71426

**Published:** 2024-10-14

**Authors:** Ishika Chaudhary, Vinay Prasad S, Ganavi Y P, Navikala K, Manjunath P R, Chitra Selvan, Pramila Kalra

**Affiliations:** 1 Endocrinology, Ramaiah Medical College, Bengaluru, IND; 2 Endocrinology and Diabetes, Ramaiah Medical College, Bengaluru, IND; 3 Biochemistry, Ramaiah Medical College, Bengaluru, IND; 4 Endocrinology and Metabolism, Ramaiah Medical College, Bengaluru, IND

**Keywords:** basal metabolic rate, body composition, insulin resistance, irisin, metabolic syndrome, pcos, polycystic ovarian syndrome

## Abstract

Introduction

Polycystic ovarian syndrome (PCOS) is a prevalent endocrine-metabolic disorder impacting women of reproductive age, often manifesting during adolescence. This study aimed to determine serum irisin levels in subjects with PCOS compared to healthy controls and explore the correlation between irisin levels and body composition indices.

Methods

A cross-sectional study was conducted at a tertiary care hospital. Thirty-three women with PCOS and 32 healthy, age-matched controls were recruited. Relevant socio-demographic and clinical data were collected, and body composition was analyzed using bioelectrical impedance analysis (BIA). Serum irisin levels were measured using enzyme-linked immunosorbent assay (ELISA), and statistical analysis was performed using CoGuide software (Evidencian Research Associates, Bengaluru, India).

Results

PCOS subjects exhibited significantly higher levels of clinical features associated with hyperandrogenism, including elevated systolic (SBP) and diastolic blood pressure (DBP), body mass index, waist-hip ratio, total cholesterol, low-density lipoproteins (LDL), serum testosterone, body fat percentage, subcutaneous fat percentage, visceral fat percentage, and fat mass index, along with lower high-density lipoprotein levels and lean mass percentage, compared to controls. Serum irisin levels were significantly higher in PCOS cases (10.82 (8.5-14.31) ng/mL) than in controls (2.57 (2.19-4.65) ng/mL). There was a moderate positive correlation between serum irisin and SBP, DBP, hirsutism, total cholesterol, LDL, visceral fat, and serum testosterone levels. Weak correlations were observed between serum irisin and other body fat indices. Subjects with metabolic syndrome exhibited higher irisin levels and significantly elevated body composition indices, including body fat percentage, visceral fat percentage, fat mass index, and fat-free mass index, compared to those without metabolic syndrome.

Conclusions

PCOS subjects have elevated serum irisin levels, which correlate positively with clinical and biochemical hyperandrogenism, total cholesterol, LDL, and visceral fat. These findings suggest that irisin may play a role in the metabolic and hormonal dysregulation observed in PCOS.

## Introduction

Polycystic ovarian syndrome (PCOS) is a prevalent endocrine-metabolic disorder impacting women of reproductive age, often manifesting during adolescence. Reports indicate that the community prevalence of PCOS among Indian adolescents is 9.13% [1]. Sedentary lifestyles and poor diets, which lead to increased rates of overweight and obesity, are responsible for the rise in PCOS cases. PCOS is characterized by various metabolic consequences beyond reproductive health, such as increased risks of obesity, insulin resistance (IR), type 2 diabetes mellitus (T2DM), and early arteriosclerosis [2].

Researchers have suggested that the dysregulated production of certain adipokine hormones may play a role in the pathogenesis of PCOS, given the propensity of PCOS patients to become overweight or obese and develop IR [3]. Irisin, a myokine released from skeletal muscle, has recently been investigated as a possible indicator of IR in PCOS-affected individuals. Fibronectin type III domain-containing protein 5 (FNDC5) is the source of irisin produced by an unidentified protease and is found in muscle, heart, liver, and adipose tissue [4]. Research indicates that physical activity promotes the expression of PPARγ coactivator-1 (PGC1) and FNDC5 in skeletal muscle, leading to the production of thermogenic cells that contribute to the prevention of obesity and metabolic syndrome (MetS). Irisin also plays a role in the browning of adipose tissue [5] and enhances glucose uptake in differentiated skeletal muscle cells by facilitating the translocation of GLUT4 to the plasma membrane [6]. Elevated serum irisin levels have been associated with increased energy expenditure, weight loss, decreased IR, and lower serum levels of total cholesterol, triglycerides, and free fatty acids [7].

Current studies present inconsistent findings regarding irisin levels in women with PCOS, with some indicating higher levels compared to healthy controls and others suggesting the opposite. Clarifying the role and presence of irisin among women with PCOS is critical. Irisin is involved in lipid metabolism, yet its relationship with obesity remains contentious. Additionally, there is no definitive evidence on whether irisin levels correlate with improvements in glucose homeostasis and insulin sensitivity, thus making the connection between serum irisin and body fat indices debatable. As a result, the current study aims to measure irisin levels in PCOS women and healthy controls, as well as investigate its relationship with body composition indices.

## Materials and methods

This cross-sectional study was conducted in the Department of Endocrinology at a tertiary care hospital between June 2022 and July 2024. The institutional ethical committee at Ramaiah Medical College and Hospital provided ethical approval (MSRMC/EC/SP-14/04-2022). We collected data from subjects who visited the department, briefing them about the study's objectives and procedures in a language they could understand. Informed consent was subsequently obtained from all participants. The privacy and confidentiality of participants were rigorously upheld, with their identities being anonymized to ensure confidentiality.

Euthyroid subjects between 18 and 40 years, diagnosed with PCOS based on Rotterdam criteria [8] and not receiving any drug treatment for PCOS in the preceding three months, were selected as cases. Healthy, age-matched women with regular menstrual cycles and no clinical manifestations of hyperandrogenism comprised the controls.

A comprehensive history was obtained through an interview schedule that collected socio-demographic information. Exclusion criteria were determined based on the patient's history and clinical examination. Women who were pregnant or lactating and subjects with androgen excess due to conditions such as Cushing's syndrome, hyperprolactinemia, congenital adrenal hyperplasia, glucocorticoid resistance, adrenal or ovarian tumors, or any other endocrinopathies were excluded. Additionally, individuals with galactorrhea, diabetes mellitus, hypertension, coronary artery disease, acute or chronic infections, known malignancies, or those taking oral contraceptives, antiandrogen therapy (within the past six months), lipid-lowering agents, metformin, pioglitazone, other oral antidiabetic drugs, or chronic steroid therapy were also excluded from both groups.

Clinical examinations were conducted according to standard protocols, including measurements of height, weight, BMI, waist circumference, and waist-hip ratio (WHR). We measured hirsutism using the modified Ferriman-Gallaway score [9]. Both cases and controls underwent evaluation using a bioelectrical impedance analyzer (BIA) to determine total body fat, subcutaneous fat, visceral fat, lean mass, and skeletal mass. Additionally, a standard ultrasound examination of the abdomen and pelvis was performed.

Samples of venous blood were obtained of both cases and controls following an overnight fast of eight hours and analyzed for fasting blood glucose, thyroid-stimulating hormone (TSH), testosterone, HbA1c, fasting lipid profile, and serum irisin levels. Serum testosterone was measured using a chemiluminescent immunoassay (CLIA).

Serum irisin was measured by using enzyme-linked immunosorbent assay (ELISA) kits (GENLISA from KRISHGEN Biosystems, Mumbai, India, catalog number: KBH3253). The intra‑ and inter‑assay coefficients of variation were <10% and <12%, respectively, with an assay sensitivity of 1.8 ng/mL, and were analyzed by using an ELISA reader at 450 nm.

National Cholesterol Education Program (NCEP) Adult Treatment Panel III (ATP III) criteria were used to define the presence of MetS [10].

The following formulas are used to calculate the fat-free mass index (FFMI) and fat mass index (FMI), along with fat-free mass (FFM) and fat mass calculations based on body fat percentage [11]:

FFMI is calculated as FFM in kilograms divided by height in meters squared: \[ \text{FFMI} = \frac{\text{FFM (kg)}}{\text{height (m)}^2} \] FMI is similarly calculated as fat mass (FM) in kilograms divided by height in meters squared: \[ \text{FMI} = \frac{\text{FM (kg)}}{\text{height (m)}^2} \] FFM is determined using the formula: \[ \text{Fat-free mass} = \text{weight (kg)} \times \left(1 - \frac{\text{body fat (%)}}{100}\right) \] Fat mass is calculated as: \[ \text{Fat mass} = \text{weight (kg)} \times \frac{\text{body fat (%)}}{100} \]

Statistical analysis

The sample size was determined using data from a previous study [12], which reported mean irisin levels of 11.1 ng/mL in individuals with PCOS and 8.3 ng/mL in the control group. Based on a 95% confidence interval and 80% statistical power, the minimum required sample size was calculated to be 33 participants per group.

The parametric data are presented as mean ± standard deviation, while the non-parametric data are presented as median (interquartile range). The intra-group comparison was done by using the Mann-Whitney U test and unpaired t‑test for non‑parametric data and parametric data, respectively.

Spearman’s and Pearson’s correlation coefficients were used to analyze the correlation between different parameters for non-parametric and parametric data, respectively. The primary outcome variables are serum irisin and serum testosterone, while the explanatory variables are body composition indices (body fat, WHR, lean mass, FMI, FFMI) and clinical features of PCOS (hirsutism, oligomenorrhea, menorrhagia, secondary amenorrhea, androgenic alopecia, acne, and MetS). Data entry and analysis were performed using CoGuide software 2.0 (Evidencian Research Associates, Bengaluru, India) [13].

## Results

In the present study, a total of 65 samples were analyzed, of which 33 were of women with PCOS and 32 were of women without PCOS. They were only age-matched, with a mean age of 26.48 ± 4.64 years in cases and 24.72 ± 3.12 years in controls (p = 0.117). However, there were significant statistical differences in BMI, WHR, weight, and BP measurements among cases and controls, as shown in Table 1. Only seven (21.9%) women without PCOS and 21 PCOS women (63.7%) were diagnosed with MetS.

**Table 1:** Demographic parameters of women with and without PCOS PCOS: Polycystic ovarian syndrome; BP: Blood pressure; BMI: Body mass index; FBS: Fasting blood sugar; HbA1C: Glycated hemoglobin; HDL: High-density lipoprotein; LDL: Low-density lipoprotein; TyG index: Triglyceride-glucose index; MetS: Metabolic syndrome * Non-parametric variables are expressed as median (interquartile range (IQR)) and parametric variables are expressed as mean ± SD.

Parameter	Cases (N=33)	Controls (N=32)	p-value
Age (years)	26.48 ± 4.64	24.72 ± 3.12	0.117
Systolic BP (mmHg)*	131 (123-134)	120 (108.5-126)	0.001
Diastolic BP (mmHg)*	86 (77-90)	70 (60-80)	0.001
Anthropometry			
BMI (kg/m^2^)	26.98 ± 4.71	23.21 ± 3.25	<0.001
Weight (kg)	66.72 ± 13.17	57.85 ± 7.45	<0.001
Waist-hip ratio	0.85 ± 0.05	0.79 ± 0.05	<0.001
Biochemical parameters			
FBS (mg/dL)*	100 (90.5-105)	96.5 (91-102.7)	0.217
HbA1C (%)*	5.3 (5.2-5.7)	5.25 (5.02-5.4)	0.272
Total Cholesterol (mg/dL)	168.12 ± 29.71	151.44 ± 25.43	0.022
HDL (mg/dL)	40.97 ± 6.57	46.69 ± 8.81	0.006
LDL (mg/dL)	108.38 ± 24.44	89.84 ± 23.59	0.002
Triglycerides (mg/dL)*	120 (81-153)	102.5 (85.2-135.7)	0.351
Total Testosterone (ng/dL)*	40.4 (36.2-48.75)	32.55 (26.8-38.7)	0.001
Serum Irisin (ng/mL)*	10.82 (8.5-14.31)	2.57 (2.19-4.65)	<0.001
TyG index*	4.56 (4.50-4.72)	4.58 (4.50-4.69)	0.958
Body composition indices			
Body fat (%)	41.49 ± 7.36	36.68 ± 6.33	0.003
Subcutaneous fat (%)	31.22 ± 5.41	27.63 ± 4.08	0.004
Visceral fat (%)	11.33 ± 2.89	9.63 ± 2.57	0.017
Lean mass (%)	46.99 ± 9.01	52.57 ± 7.38	0.010
Fat-free mass index	16.46 ± 2.21	15.48 ± 1.59	0.066
Fat mass index	17.99 ± 5.77	13.67 ± 4.05	0.001
Metabolic syndrome			
MetS present	21 (63.6%)	7 (21.9%)	<0.001

Biochemical parameters such as total cholesterol and low-density lipoproteins (LDL) were significantly higher and high-density lipoproteins (HDL) were significantly lower in women with PCOS; however, fasting blood sugar (FBS) and glycated hemoglobin (HbA1C) were similar between the two groups (Table 1).

Total testosterone (40.4 (36.2-48.75) vs. 32.55 (26.8-38.7) ng/dL, P<0.001) and serum irisin (10.82 (8.5-14.31) vs. 2.57 (2.19-4.65) ng/mL, P<0.001) levels were significantly higher in cases as compared to controls (Table 1).

Body composition indices such as body fat percentage, subcutaneous fat percentage, and visceral fat percentage, FMI were higher in PCOS women, while lean mass percentage was higher in women without PCOS (Table 1). Taking into consideration the significant differences in weight across the study groups, the FFMI was shown to be higher in women with PCOS. As a result, it is not a reliable method for determining the body composition index.

As expected, PCOS subjects had higher and more pronounced clinical hyperandrogenism in the form of hirsutism, acne, and oligomenorrhoea as compared to controls, as depicted in Table 2.

**Table 2:** Clinical features of subjects with and without PCOS PCOS: Polycystic ovarian syndrome

Clinical feature	Cases (N=33)	Controls (N=32)	p-value
Hirsutism	22 (66.7%)	4 (12.5%)	<0.001
Oligomenorrhea	17 (51.5%)	0	<0.001
Menorrhagia	4 (12.1%)	0	0.060
Secondary amenorrhea	10 (30.3%)	0	0.001
Androgenic alopecia	8 (24.2%)	0	0.003
Acne	20 (60.6%)	10 (31.3%)	0.016

The bivariate correlation analysis among PCOS subjects demonstrated a significant positive correlation between serum irisin and systolic blood pressure (SBP) (r = 0.429, P = 0.013), diastolic blood pressure (DBP) (r = 0.345, P = 0.049), total cholesterol (r = 0.530, P = 0.002), LDL (r = 0.394, P = 0.023), serum testosterone (r = 0.356, P = 0.042), and visceral fat (r = 0.389, P = 0.025). Even though serum irisin had a positive correlation with BMI, WHR, body fat percentage, subcutaneous fat percentage, FMI, and FFMI, and a negative correlation with HbA1C, HDL, and lean mass percentage, it was not statistically significant as depicted in Table 3.

**Table 3:** Correlation of irisin with other parameters in subjects with PCOS (N = 33) PCOS: Polycystic ovarian syndrome; r: Spearman’s rank correlation coefficient; WHR: Waist-hip ratio; BMI: Body mass index; BP: Blood pressure; HbA1C: Glycated hemoglobin; LDL: Low-density lipoprotein; HDL: High-density lipoprotein

Parameters	r	p-value
WHR	0.093	0.608
BMI (kg/m^2^)	0.289	0.102
Systolic BP (mmHg)	0.429	0.013
Diastolic BP (mmHg)	0.345	0.049
Hba1c (%)	-0.11	0.952
Total cholesterol (mg/dL)	0.530	0.002
LDL (mg/dL)	0.394	0.023
HDL (mg/dL)	-0.10	0.996
Triglycerides (mg/dL)	0.300	0.09
Serum testosterone (ng/dL)	0.356	0.042
Body fat (%)	0.246	0.167
Visceral fat (%)	0.389	0.025
Subcutaneous fat (%)	0.279	0.116
Lean mass (%)	-0.065	0.719
Fat mass index	0.273	0.124
Fat-free mass index	0.129	0.475

Subjects with PCOS and controls were further classified as subjects with and without MetS in each class, and their baseline parameters were compared as shown in Table 4.

**Table 4:** Baseline parameters of subjects with and without metabolic syndrome MetS: metabolic syndrome

Cases (N=33)				Controls (N=32)		
Parameters	MetS present (N=21)	MetS​​​​​​​ absent (N=7)	p-value	MetS​​​​​​​ present (N=12)	MetS​​​​​​​ absent (N=25)	p-value
Serum irisin (ng/mL)	12.15 (9.5-19.02)	8.28 (2.9-12.6)	0.015	2.34 (2.09-4.03)	3.11 (2.22-5.49)	0.144
Serum testosterone (ng/dL)	41.5 (37.5-49.8)	36.5 (36-40.5)	0.046	29.4 (26.4-33)	34.2 (27.2-39.6)	0.254
Body fat (%)	44.20 ± 6.39	36.7 ± 6.71	0.008	45.00 ± 5.44	34.35 ± 4.31	<0.001
Lean mass (%)	45.10 ± 9.40	50.31 ± 7.52	0.072	48.13 ± 9.04	53.81 ± 6.52	0.179
Fat mass index	20.76 ± 4.96	13.15 ± 3.46	<0.001	18.66 ± 4.52	12.27 ± 2.60	<0.001
Fat-free mass index	17.36 ± 1.95	14.89 ± 1.75	0.002	14.64 ± 1.17	15.71 ± 1.63	0.096
Visceral fat (%)	12.38 ± 2.78	9.50 ± 2.11	0.004	12.52 ± 2.19	8.82 ± 2.06	<0.001
Subcutaneous fat (%)	31.95 ± 4.41	29.95 ± 6.85	0.314	31.70 ± 3.77	26.49 ± 3.44	0.002

PCOS women with MetS had significantly higher serum irisin (12.15 (9.5-19.02) vs 8.28 (2.9-12.6) ng/mL, P = 0.015), serum testosterone (41.5 (37.5-49.8) vs 36.5 (36-40.5) ng/dL, P = 0.046), body fat percentage (44.20 ± 6.39 vs. 36.7 ± 6.71, P = 0.008), visceral fat percentage (12.38 ± 2.78 vs. 9.50 ± 2.11, P = 0.004), FMI (20.76 ± 4.96 vs. 13.15 ± 3.46, P <0.001), and FFMI (17.36 ± 1.95 vs. 14.89 ± 1.75, P = 0.002) as compared to women with PCOS without MetS.

Table 5 presents the correlation between serum irisin and other parameters in women with PCOS and MetS. Bivariate correlation analysis only found a significant positive correlation with total cholesterol (r = 0.585, P = 0.005), LDL (r = 0.593, P = 0.005), and visceral fat (r = 0.538, P = 0.012) among cases with MetS.

**Table 5:** Correlation of irisin with other parameters in subjects with metabolic syndrome in PCOS patients (N=21) PCOS: Polycystic ovarian syndrome; r: Spearman’s rank correlation coefficient; WHR: Waist-hip ratio; BMI: Body mass index; BP: Blood pressure; HbA1C: Glycated hemoglobin; LDL: Low-density lipoprotein; HDL: High-density lipoprotein

Parameters	r	p-value
WHR	0.094	0.687
BMI (kg/m^2^)	0.040	0.865
Systolic BP (mmHg)	0.163	0.480
Diastolic BP (mmHg)	0.103	0.656
Hba1c (%)	0.086	0.709
Total cholesterol (mg/dL)	0.585	0.005
LDL (mg/dL)	0.593	0.005
HDL (mg/dL)	0.249	0.277
Triglycerides (mg/dL)	0.122	0.600
Serum testosterone (ng/dL)	0.057	0.805
Body fat (%)	0.032	0.892
Visceral fat (%)	0.538	0.012
Subcutaneous fat (%)	0.034	0.883
Lean mass (%)	0.223	0.330
Fat mass index	0.085	0.714
Fat-free mass index	-0.040	0.862

A correlation was found between serum irisin and hirsutism in order to determine whether or not there was any relevant association between serum irisin and clinical hyperandrogenism. According to Table 6, there was a statistically significant increase in the levels of serum irisin in women with PCOS with hirsutism.

**Table 6:** Association of serum irisin with hirsutism in cases and controls

Parameter	Cases (N=33)		p-value	Controls (N=32)		p-value
	Hirsutism present (N=22)	Hirsutism absent (N=11)		Hirsutism present (N=4)	Hirsutism absent (N=28)	
Serum irisin (ng/mL)	18.23 ± 12.99	9.94 ± 8.16	0.032	3.50 ± 2.59	4.62 ± 5.61	0.351

## Discussion

PCOS is a common endocrine-metabolic condition that is common during adolescence and in women of reproductive age. This study aimed to assess and compare serum irisin levels between individuals with PCOS and healthy controls, as well as correlate them with body composition indices. The findings indicated notable differences in serum irisin levels, body composition, and metabolic parameters between the PCOS patients and the control group. A total of 32 healthy controls and 33 PCOS women participated in the study, all of whom attended the hospital between June 2022 and July 2024.

All PCOS cases in this study were within the reproductive age range, aligning with the findings of Arunachalam et al., who investigated metabolic differences between lean and obese women with PCOS [14]. The study found that the PCOS group had significantly higher BMI, weight, and WHR compared to the controls. This is similar to what Behboudi-Gandevani et al. found, which was that women with PCOS were younger, in their reproductive years, and had higher BMIs [15]. Similarly, Chitme et al. found that PCOS patients had a higher BMI, body fat distribution, waist and hip circumference, diastolic and mean blood pressure, visceral fat, and a disproportionate increase in global fat distribution [16]. Overweight and obesity are prevalent among women with PCOS and exacerbate many of its reproductive and metabolic symptoms. Increased weight may be a result of IR, which is common in PCOS-affected women. Although the exact mechanisms underlying this are unclear, it is believed to entail abnormalities in the post-receptor phosphatidylinositol 3-kinase (PI3-K) insulin pathway.

The PCOS group had significantly higher serum irisin levels compared to age-matched controls, which aligns with studies that suggest elevated irisin, an adipomyokine linked to glucose uptake and energy expenditure, as an adaptive mechanism for the increased energy expenditure commonly associated with PCOS. It may also be hypothesized that serum irisin levels are increased in PCOS women due to the presence of irisin resistance, similar to IR, whose validity needs to be confirmed in further studies.

Studies by Sharan et al. [1], Telagareddy et al. [17], Zhang et al. [18], and Adamska et al. [8] found increased serum irisin levels, similar to the present study, whereas other research by Wang et al. [19], Abali et al. [20], and Foda et al. [3] reported either reduced or comparable irisin levels between PCOS patients and controls.

Due to the inconsistent results of current studies, more further studies are required to fully understand the role of irisin in metabolic control and its potential as a biomarker for IR in PCOS. As expected, PCOS women in this study had significantly higher cases of clinical hyperandrogenism and oligomenorrhoea compared to controls. Serum testosterone was significantly elevated in PCOS women.

Significant differences were observed in body fat percentage, FMI, FFMI, and visceral fat percentage between individuals with and without metabolic syndrome. Irisin may be implicated in conditions marked by IR, like type 2 diabetes and metabolic syndrome, according to findings by Moreno-Navarrete et al. [21]. Similarly, Pardo et al. linked elevated irisin levels with BMI, muscle mass, and adipose tissue mass [22]. Normal ovarian function depends on how endocrine factors from muscle, gut, and ovarian tissues interact with each other. Chang et al. [23] say that irisin dysregulation may contribute to the metabolic features of PCOS.

This study found a statistically significant difference in serum irisin levels between individuals with and without hirsutism in PCOS women. This suggests that irisin may play a role in the development of hirsutism in women with PCOS. Further research is needed to fully understand the mechanism behind this association and to determine if targeting irisin levels could be a potential therapeutic strategy for managing hirsutism in PCOS patients. Additionally, exploring the relationship between irisin and other phenotypic features of PCOS may provide valuable insights into the pathophysiology of the condition.

In our study, we found significantly elevated serum irisin levels in women with metabolic syndrome, along with increased levels of body fat percentage, visceral fat percentage, FMI, FFMI, and serum testosterone levels. These findings were consistent with three other studies that showed higher irisin levels in obese groups [17,18,23].

As irisin is an adipomyokine, it is secreted from both adipocytes and myocytes; the increased levels of irisin in obese women may be probably explained through this mechanism, and also the irisin resistance hypothesis may also play a role. For example, one study [23] found that women with metabolic syndrome had higher levels of irisin, which is consistent with the theory that irisin is secreted from both adipocytes and myocytes in obese individuals. Additionally, the correlation between elevated irisin levels and increased body fat percentage and visceral fat percentage further supports the idea that irisin may play a role in obesity-related metabolic dysfunction.

The findings of our study further support the existing literature on the role of irisin in obesity and metabolic syndrome. Future research may focus on investigating the mechanisms behind irisin resistance and its implications for the management of metabolic disorders.

This study identified a moderate positive correlation between serum irisin levels and body fat, FMI, FFMI, subcutaneous fat, BMI, and WHR; however, a statistically significant correlation was seen in visceral fat, serum testosterone, total cholesterol, and LDL levels in women with PCOS. These findings were similar to previous studies, which showed a positive correlation with irisin, body fat, and testosterone [17,22,23]. Similar findings were found with PCOS women with metabolic syndrome. The significant correlation between visceral fat and cholesterol levels indicates that irisin may be involved in the pathophysiology of metabolic disturbances commonly seen in PCOS.

Despite its well-structured design, the present study has few limitations. Firstly, its small sample size and hospital-based setting limit the generalizability of the findings. Secondly, the lack of BMI matching between cases and controls could potentially alter the study's results. Thirdly, the study's cross-sectional design limits the ability to establish causation between elevated serum irisin levels and PCOS or its metabolic characteristics. Finally, PCOS is a heterogeneous condition with various phenotypes. The lack of stratification based on specific phenotypes may obscure nuanced differences in metabolic parameters and serum irisin levels. While the study suggests potential roles for irisin, it does not delve into the underlying biological mechanisms. To confirm these results, larger studies are necessary.

## Conclusions

This study enhances the understanding of irisin's role in PCOS, revealing elevated serum irisin levels among patients with PCOS and metabolic syndrome, irrespective of total body fat percentage. Visceral fat positively correlates with serum irisin levels. We need more research to explore the potential of irisin as a biomarker and therapeutic target in PCOS and its associated metabolic complications.
